# High-Temperature Synthesis of High-Entropy Alloy PtPd_CoNiCu Nanoparticles as a Catalyst for the Oxygen Reduction Reaction

**DOI:** 10.3390/ijms262311504

**Published:** 2025-11-27

**Authors:** Alina Nevelskaya, Anna Gavrilova, Nikolay Lyanguzov, Mikhail Tolstunov, Ilya Pankov, Anna Kremneva, Evgeny Gerasimov, Andrey Kokhanov, Sergey Belenov

**Affiliations:** 1 Faculty of Chemistry, Southern Federal University, 7 Zorge St., Rostov-on-Don 344090, Russia; alya.nevelskaya@mail.ru (A.N.); agavrilo@sfedu.ru (A.G.);; 2 Federal Research Center the Southern Scientific Center of the Russian Academy of Sciences (SSC RAS), 41 Chekhov Ave., Rostov-on-Don 344006, Russia; miftol@yandex.ru; 3 Department of Physics, Southern Federal University, 5 Zorge St., Rostov-on-Don 344090, Russia; 4 Research Institute of Physical Organic Chemistry, Southern Federal University, 194/2 Stachki St., Rostov-on-Don 344090, Russia; ipankov@sfedu.ru; 5 Boreskov Institute of Catalysis, Ac. Lavrentieva Ave. 5, Novosibirsk 630090, Russia

**Keywords:** platinum-based electrocatalysts, high-entropy alloys, multicomponent systems, high-temperature synthesis, heat treatment, oxygen reduction reaction, durability

## Abstract

The aim of this work was high-temperature synthesis of PtPdCoNiCu/C nanoparticles with high-entropy alloy (HEA) structure as catalysts for oxygen reduction reaction. The materials were synthesized using a highly dispersed PtPd/C support, which was impregnated with Cu, Ni, and Co precursors followed by their precipitation with an alkali. Subsequently, the material was subjected to thermal treatment in a tube furnace at 600 °C for 1 h in a stream of argon containing 5% hydrogen. In combination with HRTEM, element mapping and line scan, XRD, and XPS data, these results confirm the successful synthesis of five-component PtPdCoNiCu high-entropy alloy nanoparticles on the surface of the carbon support. The obtained materials are characterized by a high electrochemical surface area of up to 63 m^2^/g(PGM), as determined by hydrogen adsorption/desorption and CO-stripping, and a high specific oxygen reduction reaction (ORR) activity of approximately 269 A/g(PGM) at 0.9 V vs. RHE. The synthesized material demonstrated outstanding stability, as confirmed by an accelerated stress test of 10,000 cycles. After the test, the electrochemical surface area decreased by only 12%, while the catalytic activity for ORR even increased. The proposed synthetic strategy opens a new pathway for obtaining promising highly stable five-component HEA nanoparticles of various compositions for application in catalysts.

## 1. Introduction

The transition to renewable energy sources requires the development of a comprehensive set of technologies for energy storage and transmission. Hydrogen-based systems are among the most promising solutions for energy storage and transportation. In this context, low-temperature fuel cells (LTFCs) efficiently convert hydrogen’s chemical energy into electricity. Catalysts are critical LTFC components, as they accelerate the hydrogen oxidation reaction (HOR) at the anode and the oxygen reduction reaction (ORR) at the cathode. The latter reaction, however, proceeds with a high overpotential [1]. To date, the most used catalysts in LTFCs are platinum or platinum-alloy nanoparticles supported on highly dispersed carbon materials. Current research is focused on finding ways to enhance the ORR activity of these catalysts [2], reduce their cost, and improve their stability [3,4,5]. These improvements are essential for lowering the cost of LTFCs and extending their lifespan, which will facilitate the wider adoption of such devices and advance the development of the hydrogen economy.

Among a wide range of approaches to improving the characteristics of low-temperature fuel cell (LTFC) catalysts, the use of platinum group metal-based high-entropy alloy nanoparticles supported on carbon materials represents a novel and relatively underexplored strategy [6,7], as discussed in works on PtPdFeCoNi HEA nanocatalysts and related systems [8,9,10,11].

Such systems contain five or more metallic components in equimolar ratios, which form a solid-solution alloy structure [12]. Due to the high-entropy effect and the presence of numerous different active sites and strain effects on the surface of HEA nanoparticles, such systems are promising for applications in catalysis [13,14,15], particularly for the ORR [15,16].

The synthesis of such HEA systems on carbon support and the confirmation of their structure formation is a complex challenge that requires a comprehensive approach [11,14,17].

For the synthesis of HEA nanoparticles, methods like those used for metallic nanoparticles are employed [11,17]. However, in this case, besides the need to form small-sized nanoparticles with a narrow size distribution and their uniform dispersion on the carbon support surface, it is also crucial to achieve the formation of nanoparticles consisting of a homogeneous alloy from many metals. To facilitate this, metals with the same crystal structure type and similar lattice parameters are typically used [18] to promote the formation of a homogeneous alloy with an equimolar metal ratio.

For the synthesis of HEA nanoparticles, both “top-down” and “bottom-up” approaches are used [7,14].

In the “top-down” approach, nanoparticles are separated from a bulk HEA sample via dispersion. The advantage of this method is its simplicity [19]. However, similar to the synthesis of metallic nanoparticles, this method cannot yield sufficiently small nanoparticles and offers limited control over the size and shape of the resulting nanoparticles. Furthermore, such methods are typically energy-intensive, and the synthesis process leads to changes in surface chemistry and significant alterations in the physical and chemical properties of the nanomaterials [20].

The “bottom-up” approach involves the formation of nanoparticles from atoms, typically through the reduction of corresponding metal ions during chemical synthesis. Various methods are used to fabricate HEA nanoparticles, including wet-synthesis (hydrothermal and solvothermal); local thermal shock methods (microwave, carbothermal, and ultrasonic); co-deposition from metal precursors (such as electrodeposition, spraying, and magnetron sputtering); and sol–gel methods and pyrolysis of metal precursors [21,22,23,24,25].

Wet-synthesis methods allow for control over the composition and characteristics of the resulting nanoparticles by selecting specific reducing agents, tuning the medium composition (solvents, surfactants), and adjusting the nanoparticle formation conditions (temperature, heating rate, stirring conditions, etc.). All these factors provide extensive opportunities to influence the composition and structure of the resulting HEAs, and consequently, their catalytic activity.

Another promising method for synthesizing multicomponent nanoparticles is the impregnation of Pt/C catalysts followed by reduction in an inert atmosphere with hydrogen addition [26]. In the case of multicomponent nanoparticles, it is advisable to use not Pt/C but PtM/C materials, such as PtPd/C or PtRh/C, as the foundation at the initial synthesis stage.

It is important to note that during high-temperature synthesis, the characteristics of the resulting catalyst are significantly influenced by both the heat treatment conditions [27,28], primarily temperature [29,30,31], and the composition of the reducing atmosphere, as well as the characteristics of the initial Pt/C or PtM/C material and the impregnation method used.

Furthermore, in some cases, various types of post-treatment are employed to improve the catalyst structure and alter its surface composition [28], such as thermal treatment in a reducing atmosphere or acid treatment. During high-temperature post-treatment, the degree of alloying in the obtained multicomponent systems can be increased through interdiffusion of metals, leading to the formation of a homogeneous alloy [32]. On the other hand, under certain conditions, the alloy structure can be disrupted due to metal segregation processes. This fact necessitates the optimization of high-temperature treatment conditions, including temperature, duration, and atmosphere composition, since the adsorption of certain components can lead to adsorption-induced selective segregation of components.

Acid treatment leads to the selective dissolution of non-noble components from the nanoparticle surface and the dissolution of non-noble components that did not in-corporate into the alloy [33,34]. Such treatment can result in the formation of core–shell structures, where the shell consists of platinum group metals and the core is a HEA. Due to electronic effects, where the core influences the electronic state of the nanoparticle surface, the activity of such materials can be higher than that of pure metals while maintaining enhanced stability, owing to the protective effect of the platinum group metal shell.

Research on platinum-group metal-based HEAs for the ORR is still at an early stage, particularly for systems doped with various d-metals, where the focus remains on accumulating fundamental experimental data. Currently, Pt-based HEAs represent the most studied class of ORR catalysts [35], typically synthesized by combining platinum with other metals that possess similar lattice parameters and crystal structures to ensure miscibility. In this study, we selected PtPd-based systems as a foundation, alloying them with the d-metals most commonly used for platinum doping: Co, Ni, and Cu [36,37,38,39,40]. This strategic selection of elements is anticipated to facilitate the formation of a single-phase alloy with a stable face-centered cubic (FCC) structure.

Thus, the aim of this work was to synthesize PtPdCoNiCu HEA nanoparticles supported on a Vulcan XC-72 carbon carrier using the impregnation of a PtPd/C material with salts of the corresponding metals, followed by high-temperature treatment at 600 °C in an inert atmosphere with a 5% H_2_ additive, and to study the composition, structure, stability, and oxygen reduction reaction activity of the resulting catalysts.

## 2. Results

### 2.1. Composition and Structure of the PtPd_CoNiCu/C Catalyst

**Figure 1:**
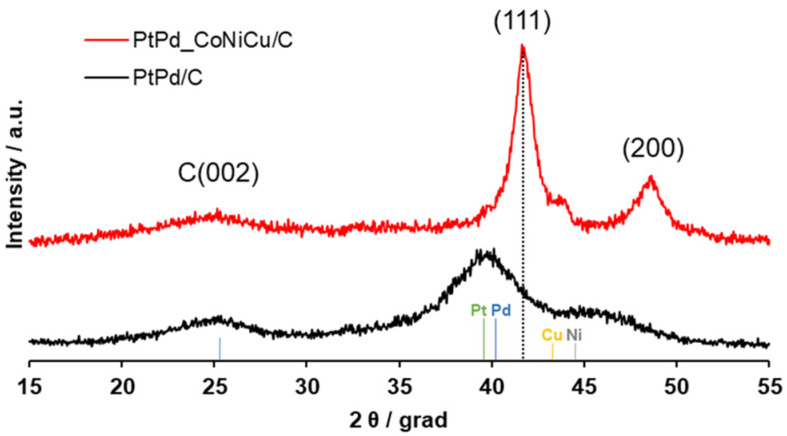
X-ray diffraction patterns of the PtPd/C base material and the PtPd_CoNiCu/C catalyst derived from it.

The PtPd/C material obtained at the first synthesis stage contains 28.8 wt% of metals, as determined by thermogravimetric analysis, and exhibits an atomic composition of PtPd_0.9_ based on elemental analysis data (Table 1). This finding shows good correlation with the metal content expected from the precursor loading. According to XRD analysis, the synthesized PtPd/C material (Figure 1) consists of a carbon phase and a metallic phase with a face-centered cubic crystal structure, having a lattice parameter of approximately 3.941 Å. It should be noted that based solely on the position of the maximum metallic phase reflection, it is not possible to unambiguously distinguish between Pt-Pd alloy and overlapping individual phases of platinum (3.924 Å) and palladium (3.890 Å). This ambiguity arises because the lattice parameters of these metals are very similar and the diffraction peaks exhibit significant broadening [40,41], further complicated by the effect of nanoparticle size on the lattice parameter [42]. The crystallite size, calculated from the full width at half maximum (FWHM) value for the PtPd/C material under the assumption of a single metallic phase, was approximately 1.9 nm. Thus, a finely dispersed PtPd/C material with small crystallite size was successfully synthesized at the first stage, making it suitable for the subsequent preparation of the PtPd_CoNiCu/C catalyst.

The impregnation of the PtPd/C material with metal precursors, followed by their precipitation with alkali and thermal treatment in a reducing atmosphere (Figure 1), yielded a PtPd_CoNiCu/C catalyst with a total metal content of 36.3 wt % and a composition of Pt_16_Pd_17_Co_24_Ni_22_Cu_21_. Considering this composition, the platinum group metal (PGM) content in this catalyst is 19.9 wt % (Table 1), which is close to the value expected from the precursor loading.

The powder diffraction pattern of the resulting PtPd_CoNiCu/C catalyst shows a shift in the metallic phase reflections compared to the base PtPd/C material (Figure S1). This shift is associated with the incorporation of Ni, and/or Co, and/or Cu into the alloy, leading to a decrease in the crystal lattice parameter from 3.941 Å for PtPd/C to 3.752 Å for PtPd_CoNiCu/C. It is noteworthy that the theoretical lattice parameter for a material with an equal atomic fraction of all components (Pt_20_Pd_20_Co_20_Ni_20_Cu_20_) is 3.701 Å, which is reasonably close to the lattice parameter of the obtained sample and indicates a high degree of component alloying in the synthesized catalyst. Furthermore, the FWHM of the metallic phase reflections decreases compared to the PtPd/C material. The crystallite size calculated using the Scherrer equation was about 5.5 nm. This increase in size is attributed both to the incorporation of metal atoms into the PtPd nanoparticles and to nanoparticle coarsening during thermal treatment.

Thermogravimetric analysis/differential scanning calorimeter (TGA/DSC) analysis provides a comprehensive characterization of Pt-based catalysts. This method not only determines the total metallic phase content from the mass residue following complete oxidation of the carbon support but also quantifies key combustion parameters, such as the onset temperature, maximum reaction rate temperature, and the temperature range of intensive oxidation. Furthermore, it enables investigation of the specific characteristics of this process as a function of the composition and microstructure of the deposited metal nanoparticles [43,44,45]. According to the DSC results (Figure S2a) for the PtPd_CoNiCu/C material, the combustion of the carbon support occurs at lower temperatures (DSC combustion maximum at 470 °C) compared to the PtPd/C material used as a base material in the first stage of synthesis (DSC combustion broad peak with a maximum of about 480 °C). It should be noted that this temperature (470 °C) is closest to the combustion maximum of the PtCu/C material (469 °C) [46], while the combustion maximum for the PtNi/C and PtCo/C materials is higher (581 °C and 513 °C, respectively), compared to the five-component PtPd_CoNiCu/C material. The combustion termination temperature for the PtPd_CuCoNi/C material is approximately 20 °C lower compared to the PtPd/C reference. The most notable differences in the combustion profiles of the PtPd_CoNiCu/C and PtPd/C materials include a narrower temperature range for the combustion of the PtPd_CoNiCu/C catalyst.

Temperature-programmed reduction (TPR) analysis was used to identify the temperature ranges of various reduction processes by monitoring hydrogen uptake, which is consumed in redox reactions, as a function of temperature. In this case, the reduction of the PtPd_CoNiCu_Alk/C material was studied. This sample was prepared by depositing Co, Ni, and Cu hydroxides via alkali precipitation onto a PtPd/C material, followed by thermal treatment in an atmosphere identical to that used during the furnace synthesis.

Analysis of the TPR profile for the PtPd_CoNiCu_Alk/C sample (Figure S2b) reveals that the reduction of Co, Ni, and Cu oxides/hydroxides to their metallic states occurs in the temperature range of 250–300 °C, with a total hydrogen consumption of 2.7 mmol/g (cat). In contrast, the TPR profile of the initial PtPd/C material (Figure S2c) shows no such hydrogen uptake peaks in this region, with a significantly lower total hydrogen consumption of only 0.6 mmol/g (cat).

**Figure 2:**
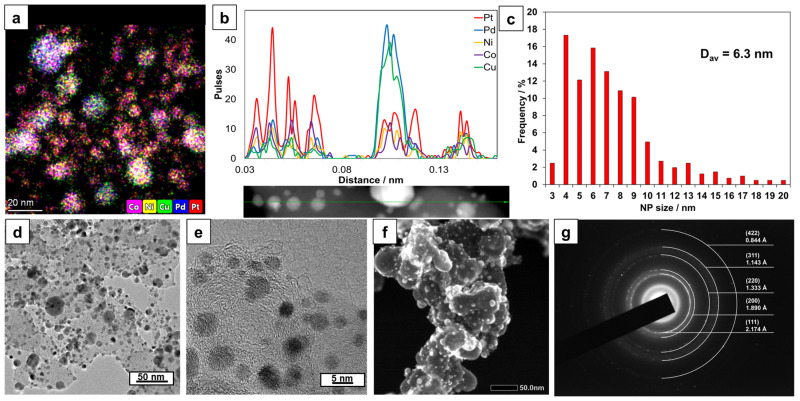
Characterization of PtPd_CoNiCu/C catalyst: (**a**) elemental mapping; (**b**) elemental line scan profile; (**c**) particle size distribution histogram; (**d**,**e**) TEM images at different magnifications; (**f**) SEI micrograph; (**g**) SAED image.

Transmission electron microscopy (TEM) analysis of the synthesized PtPd_CoNiCu/C catalyst confirmed the formation of five-component PtPdCoNiCu nanoparticles on the carbon support surface. Elemental mapping of a catalyst region (Figure 2a) revealed the presence of metallic nanoparticles containing signals from all five metals (Figure S3). Line-scanning analysis (Figure 2b) further verified the formation of these five-component nanoparticles. However, it should be noted that the composition of the investigated particles is heterogeneous. According to the line-scan data, smaller nanoparticles (3–5 nm) contain a relatively high proportion of platinum, whereas larger nanoparticles (around 10 nm) are predominantly composed of Pd and Cu. A similar trend is observed in the elemental maps of specific surface areas (Figure S3).

The heterogeneity of the nanoparticle composition is further supported by the determination of interatomic distances for individual particles using high-resolution TEM (HRTEM) (Figure S4). Selected area electron diffraction (SAED) (Figure 2g) allowed for a precise determination of the crystal lattice parameter for the metallic nanoparticles in the selected area. For the PtPd_CoNiCu/C catalyst, this parameter was measured at 3.766 Å, which is in good agreement with the value determined by XRD (Table 1). This consistency indicates the formation of PtPd alloy with Co, Ni, and Cu. It is important to note that the local SAED pattern shows no reflections corresponding to oxide phases of Cu, Ni, or cobalt, consistent with the XRD data for the PtPd_CoNiCu/C catalyst (Figure 1).

Analysis of the TEM images (Figure 2d,e) enabled the determination of individual metal nanoparticle sizes and construction of a size distribution histogram (Figure 2c), revealing an average nanoparticle size of 6.3 nm. The particle size distribution is complex, predominantly featuring particles between 4 and 9 nm with a broad distribution, alongside larger nanoparticles ranging from 10 to 20 nm. Notably, the TEM-derived average particle size exceeds the XRD-derived crystallite size (Table 1), which is typical for platinum-containing catalysts [47]. This discrepancy may originate from methodological differences in size determination and/or the presence of an X-ray amorphous shell surrounding the nanoparticles.

HRTEM analysis shows that most nanoparticles, regardless of size, feature a darker, well-crystallized core surrounded by a lighter amorphous shell (Figure S5) approximately 2–3 nm thick. We propose this shell consists of amorphous carbon formed during thermal treatment, consistent with reports in literature [48,49,50,51,52]. Such structural features have been shown to significantly enhance the stability of platinum-containing catalysts [53,54].

Analysis of specific surface areas using a combination of different scanning transmission electron microscopy (STEM) modes (Figure S6) allows for characterization of the local morphology (via secondary electron imaging (SEI) and the distribution of nanoparticles on the surface and within the volume of the spherical carbon particles. This is possible because BF-STEM and HAADF detect all metallic nanoparticles, while SEI and backscattered electron imaging (BEI) primarily reveal nanoparticles located on the external surface of the carbon support. The analysis of surface regions using various microscopy modes—BF-STEM, HAADF, SEI, and BEI (Figure S6)—further enables assessment of the surface topography and the distribution of nanoparticles across the surface of the spherical carbon particles. Examination of multiple surface areas demonstrated a uniform distribution of metal nanoparticles across the surface of the spherical carbon support particles.

In summary, the PtPd_CoNiCu/C material exhibits significant heterogeneity in both nanoparticle composition and size. This heterogeneity suggests the presence of numerous diverse active sites, which several studies indicate could be advantageous for catalytic versatility [13,14,15].

Thus, in combination with the elemental analysis and XRD data, these TEM results confirm the successful synthesis of five-component PtPdCoNiCu high-entropy alloy nanoparticles on the carbon support via high-temperature synthesis in a reducing atmosphere using a PtPd/C catalyst as a precursor.

**Figure 3:**
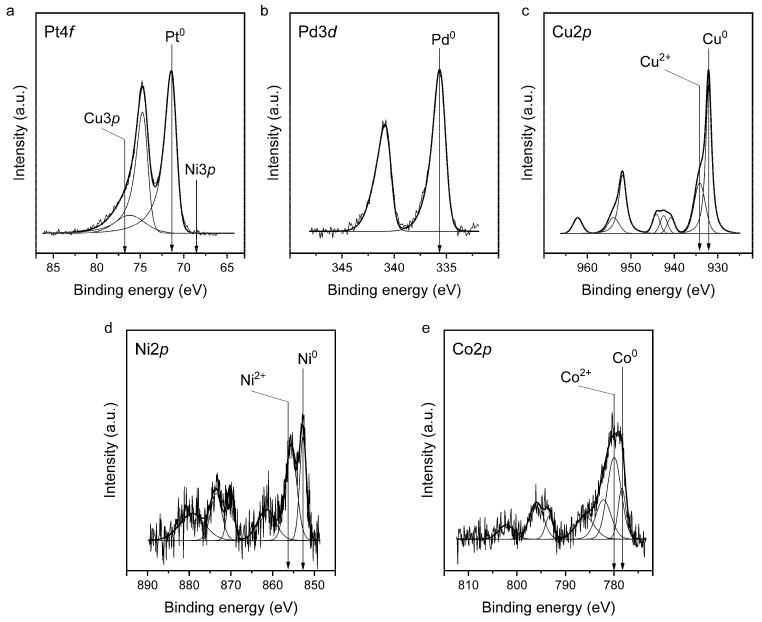
Pt *4f* (**a**), Pd *3d* (**b**), Cu *2p* (**c**), Ni *2p* (**d**) and Co *2p* (**e**) core-level spectra of the PtPd_CoNiCu/C catalyst with a high-entropy alloy structure. All the spectra are normalized to the total intensity of the corresponding C *1s* spectrum.

XPS results provide information on the surface composition and chemical state of atoms in the nanoparticles. Let us examine the spectra of each element (Figure 3) in more detail. The Pt 4f spectra exhibit Pt 4*f_7/2_—*Pt 4*f_5/2_* doublet where the spin–orbit splitting is 3.33 eV. In the case of the studied PtPd_CoNiCu/C catalyst (Figure 3a), the Pt 4f spectrum is fitted by a Pt 4*f_7/2_—*Pt 4*f_5/2_* doublet with the Pt 4*f_7/2_* binding energy of approximately 71.2–71.3 eV which corresponds to platinum in the metallic state [55,56]. It should be noted that the spectral regions of Cu 3*p* and Ni 3*p* partially overlap with the Pt 4*f* spectral region. However, in this sample, the intensity of the Cu 3*p* and Ni 3*p* spectra is significantly lower compared to the intensity of the Pt 4*f* peaks due to the low relative concentration of copper and nickel atoms on the sample surface; furthermore, the the cross-section for Cu *3p* and Ni *3p* line spectra is very small.

It is known that the Pd 3*d* spectrum of palladium consists of a Pd 3*d*_5/2_–Pd 3*d*_3/2_ doublet, with a spin–orbit splitting of 5.26 eV. The spectra of the PtPd_CoNiCu/C catalyst (Figure 3b) show a Pd 3*d*_5/2_–Pd 3*d*_3/2_ doublet with the Pd 3*d*_5/2_ binding energy of 335.5 eV, which corresponds to palladium in the metallic state [57,58,59]. This value is slightly higher than the Pd 3*d*_5/2_ binding energy for bulk metallic palladium. Such a small increase in binding energy can arise due to the interaction of small Pd clusters with the conductive carbon support [58]. That is, for small clusters, there is a possibility of a binding energy shift due to interatomic charge transfer to the support.

The Cu 2*p*, Ni 2*p*, and Co 2*p* spectra consist of the 2*p_3/2_*_2p*_1/2_* doublets, with an area ratio of approximately 2:1. The spin–orbit splitting for metallic Cu, Ni, and Co is 19.8, 17.3, and 15.0 eV, respectively, and can vary slightly depending on the oxidation state.

In the Cu 2*p_3/2_* spectrum of the PtPd_CoNiCu/C sample (Figure 3c) contains two spin–orbital doublets with the Cu 2*p_3/2_* binding energies around 932.1 and ~934.2 eV, along with several satellite peaks. The high Cu 2*p_3/2_* binding energy of the doublet at 934.2 eV and intense shake-up satellites indicate the presence of Cu^2+^ species. To identify the second doublet, we measured the Auger-parameter, which was 1850.6 eV, indicating that copper was in the metallic state. Based on the obtained XPS data, it can be inferred that copper in the PtPd_CoNiCu/C sample exists in a mixed state of Cu^2+^ and Cu^0^ (see Table S1).

The Ni 2*p* spectrum of the PtPd_CoNiCu/C sample also shows a peak with the Ni 2*p_3/2_* binding energy around 852.7 eV, corresponding to nickel in the metallic state, and a peak at 855.6 eV characteristic of Ni^2+^ compounds [60,61]. The literature [60] reports the Ni 2*p_3/2_* binding energy for nickel oxide (NiO) and nickel hydroxide (Ni(OH)_2_) of approximately 854.0 eV and 855.6 eV, respectively. It can be proposed that nickel in the PtPd_CoNiCu/C sample is present in the form of hydroxide and metallic nickel.

According to literature data, the Co 2*p_3/2_* binding energy for metallic cobalt (Co^0^) is 778.0–778.1 eV, while for cobalt in CoO and Co_3_O_4_ oxides, the reported Co 2*p_3/2_* binding energies are 780.0–780.5 eV and 779.6–780.7 eV, respectively [61,62,63]. The Co 2*p* spectrum of the PtPd_CoNiCu/C sample exhibits a peak with the Co 2*p_3/2_* binding energy around 780.0 eV with corresponding satellites, which is characteristic of Co^2+^, along with a peak at approximately 778.4 eV attributable to metallic cobalt (Co^0^), accounting for approximately 16% of the total cobalt. Thus, the PtPd_CoNiCu/C catalyst contains cobalt atoms in both Co^0^ and Co^2+^ states (see Table S1).

Based on the surface composition analysis, the successful formation of a five-component alloy can be confirmed. During the synthesis, Ni, Co, and Cu were deposited as hydroxides onto the surface of the PtPd nanoparticles. In the case of incomplete interdiffusion of atoms, the surface would have been expected to be enriched with these metals. The observed surface composition, however, indicates that significant interdiffusion has occurred, leading to alloy formation rather than a simple surface deposition.

### 2.2. Electrochemical Characteristics of the PtPd_CoNiCu/C Catalyst

**Figure 4:**
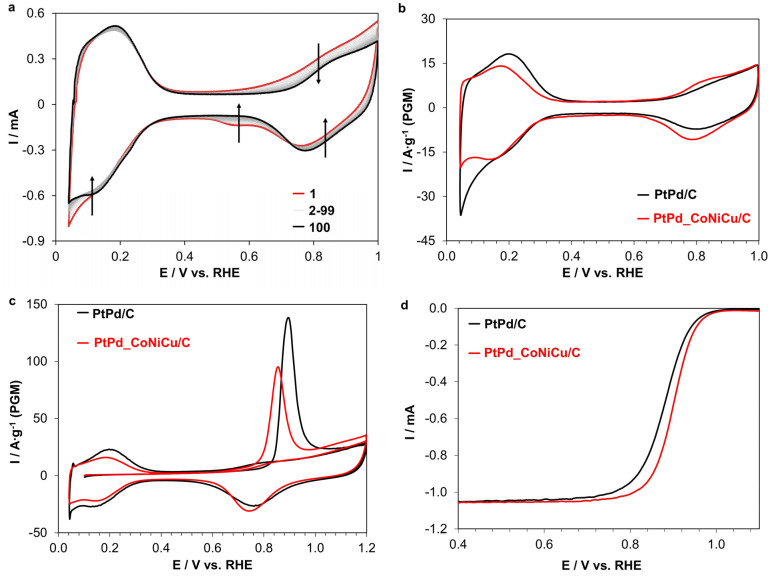
Electrochemical characterization of the PtPd/C and PtPd_CoNiCu/C catalysts: (**a**) Surface standardization (the arrow indicates the direction of the cycle from 1 to 100), (**b**) Cyclic voltammograms, (**c**) CO-stripping voltammograms, and (**d**) ORR polarization curves.

The electrochemical behavior of PtPd/C and PtPd_CoNiCu/C catalysts was evaluated in a three-electrode cell using a rotating disk electrode. Prior to electrochemical measurements, catalyst surface standardization was performed by recording 100 cyclic voltammograms (CV) at a high potential scan rate of 200 mV/s (Figure 4a). The CVs showed changes in curve shape from cycle to cycle, indicated by arrows in the figure, but by cycles 20–30 the curve shape stabilized with no further changes, indicating successful catalyst surface standardization. It should be noted that several studies report the presence of cathodic copper dissolution peaks from the alloy and metallic copper dissolution peaks during the standardization stage [64,65]. For the PtPd_CoNiCu/C catalysts, no such copper dissolution peaks were observed.

The CVs of PtPd/C and PtPd_CoNiCu/C catalysts recorded in HClO_4_ under argon atmosphere exhibit characteristic features of Pt or PtPd-based catalyst surface processes (Figure 4b) [41,46]. The CVs of the PtPd_CoNiCu/C catalyst show lower intensity of hydrogen adsorption/desorption peaks. Specifically, the electrochemical surface area (ECSA) calculated from the charge associated with hydrogen adsorption/desorption showed that for the PtPd/C catalyst the ECSA is 83 m^2^/g (PGM), while for the PtPd_CoNiCu/C catalyst the ECSA decreased by a factor of 1.3 to 63 m^2^/g (PGM) (Table 2). The relatively high ECSA value of the obtained PtPd_CoNiCu/C catalyst is most likely associated with the small nanoparticle size and high ECSA of the base PtPd/C material. This decrease in ECSA can be attributed to the increased nanoparticle size of the PtPd_CoNiCu/C catalyst after doping compared to the PtPd/C catalyst nanoparticles (from 1.9 to 5.5 nm), which reduces the ECSA value.

In addition to the data obtained from the hydrogen region of the CV, the behavior of the catalysts during the chemisorption and oxidation of carbon monoxide (CO-stripping) was studied to comprehensively characterize the surface and independently measure the ECSA. On the anodic sweep of the first cycle, both synthesized materials show a single symmetric and narrow CO oxidation peak with a maximum at approximately 898 mV for PtPd/C and 861 mV for PtPd_CoNiCu/C catalyst, respectively (Figure 4c). The PtPd/C material is also characterized by a CO monolayer oxidation peak with a larger area, confirming a greater amount of charge required for CO oxidation and, consequently, its higher ECSA compared to the PtPd_CoNiCu/C catalyst. It should be noted that for Pt/C, the CO oxidation peak maximum is typically around 0.8 V [66], indicating a change in the composition and surface properties of the five-component HEA compared to platinum, which is consistent with the XPS data. Thus, the ECSA values determined by H_ads/des_ and CO-stripping for the studied materials are similar, demonstrating good agreement between different methods for assessing the catalyst surface area in this case (Table 2).

The LSV (Figure 4d) revealed that the PtPd_CoNiCu/C catalyst outperforms the bimetallic PtPd/C template in both mass activity and specific activity in ORR. Specifically, the PtPd_CoNiCu/C material exhibits a mass activity 1.8 times greater and a specific activity 2.4 times higher than the template (Table 2). Furthermore, the half-wave potential (E_1/2_) of the PtPd_CoNiCu/C catalyst reached 0.90 V, which is 10 mV higher than that of the PtPd/C template, despite its lower ECSA. For the PtPd_CoNiCu/C catalyst, the number of electrons (n) participating in the current-generating reaction, as determined by the slope of the Koutecky-Levich plots, was found to be 3.9. This value points to a 4-electron pathway for the ORR and confirms the catalyst’s high quality. The enhanced surface activity of the PtPd_CoNiCu/C catalyst compared to the PtPd/C template confirms effective doping and the positive effect of using high-entropy nanoparticles for the ORR. A comparison with the commercial benchmark catalyst Hispec 3000 demonstrates comparable mass activity for the ORR, while the specific activity of the synthesized PtPd_CoNiCu/C catalyst is somewhat higher (Table 2). Thus, the obtained PtPd_CoNiCu/C catalyst shows good activity but requires further optimization to increase the ECSA, including through synthesis temperature adjustment.

**Figure 5:**
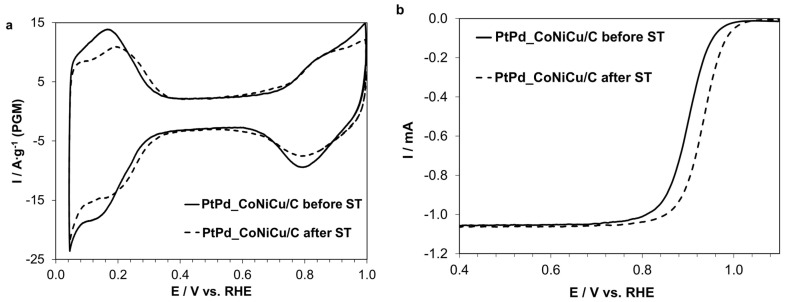
CV (**a**) and LSV (**b**) before and after stress-test of PtPd_CoNiCu/C catalyst.

To evaluate the stability of the PtPd_CoNiCu/C catalyst, an accelerated stress test (AST) was conducted in an oxygen-saturated environment. The synthesized PtPd_CoNiCu/C material demonstrated high stability in terms of ECSA (approximately 88% retention) (Figure 5a) after 10,000 cycles. Concurrently, analysis of the residual ORR activity revealed its significant increase (Figure 5b) following the AST. Moreover, the number of electrons for the material after AST remained virtually unchanged at 3.8, indicating no alteration in the ORR mechanism. It is important to note that the commercial Pt/C catalyst Hispec 3000 showed significantly worse performance under identical conditions: a 43% decrease in ECSA and a 57% decrease in ORR activity. This effect is most likely associated with an increase in the catalyst’s surface activity due to changes in its surface composition. Furthermore, the formation of an amorphous shell, observed by TEM (Figure S5), could significantly enhance the nanoparticles’ resistance to agglomeration. However, elucidating the precise reasons for this effect requires additional studies of the catalyst’s surface composition using XPS and its microstructure using TEM after stress-testing. These necessary investigations are beyond the scope of this publication and will be undertaken in the near future.

## 3. Discussion

Thus, five-component PtPdCoNiCu HEA nanoparticles on carbon support with a total metal loading of approximately 36 wt%, containing over 20 wt% platinum group metals (PGMs), and a composition of Pt_16_Pd_17_Co_24_Ni_22_Cu_21_ as determined by TXRF, were successfully synthesized via high-temperature synthesis following the impregnation of a PtPd/C precursor with Cu, Ni, and Co salts. A comprehensive suite of characterization techniques, including XRD, TEM, EDX, TXRF, TGA/DSC, and XPS, was employed to study the composition and structure of the obtained PtPd_CoNiCu/C catalyst. The formation of five-component PtPdCoNiCu HEA nanoparticles with an FCC structure was confirmed.

It is important to note that the synthesized nanoparticles are characterized by a relatively broad size distribution and compositional heterogeneity. On the other hand, this indicates the presence of many diverse active sites on the catalyst surface. HRTEM results demonstrate the formation of an amorphous carbon shell around the metal particles, which, along with the high-entropy effect, may contribute to their high stability. Despite the relatively large average crystallite size of 5.5 nm determined by XRD and a nanoparticle size of 6.3 nm from TEM, the material exhibits a high ECSA of 63 m^2^/g (for PGM) as confirmed by two independent methods. This value is only slightly lower than that of a commercial Pt/C catalyst with a significantly smaller particle size of about 3 nm. XPS results confirm the formation of an HEA, with platinum and palladium present predominantly in the metallic state, while Co, Ni, and Cu are found in both metallic and oxidized states. The surface composition of the synthesized HEA catalyst, as determined by XPS, was Pt_30_P_18_ Co_12_Ni_10_Cu_30_, which differs somewhat from the bulk composition obtained by TXRF (Pt_16_Pd_17_Co_24_Ni_22_Cu_21_, Table 1). This discrepancy between the surface and bulk compositions could indicate preferential surface segregation of platinum and copper atoms. Alternatively, it may be related to the variation in composition and size of the nanoparticles, as previously noted in the TEM data, where smaller nanoparticles were enriched in platinum and contribute more significantly to the surface signal due to their higher surface-to-volume ratio.

Nevertheless, it is important to note that the high copper content on the nanoparticle surface is a detrimental factor for the catalyst’s use in a Membrane Electrode Assembly (MEA). During operation, copper atoms can undergo selective dissolution from the catalyst surface and poison the ion-exchange membrane, leading to significant performance degradation of the MEA [67,68]. Therefore, additional post-treatment, such as acid leaching [36,69], may be required prior to MEA testing and will be the subject of further investigation.

The obtained PtPd_CoNiCu/C catalyst demonstrates good mass activity towards the ORR and a specific activity higher than that of a commercial Pt/C benchmark. Of greatest interest is its anomalously high stability. Accelerated stress testing over 10,000 cycles revealed only a minor decrease in the ECSA and a significant increase in ORR activity. This increase in activity after stress testing presumably indicates a restructuring of the catalyst nanoparticles, leading to the formation of a more optimal surface. Furthermore, the enhanced stability of the synthesized materials may be attributed to both the formation of an amorphous carbon layer (Figure S5), which protects the nanoparticles from agglomeration, and the high-entropy effect in the obtained materials [70]. However, this observation requires further detailed investigation of the catalyst’s composition and structure after stress testing, which is beyond the scope of this study.

Future work will focus on investigating the activity and stability of the obtained catalysts in a MEA and a detailed analysis of the catalyst’s composition and structure after stress-testing.

## 4. Materials and Methods

### 4.1. Synthesis

#### 4.1.1. Preparation of PtPd/C Materials

The PtPd/C precursor catalysts were synthesized via a liquid-phase reduction method, following a procedure like that described in ref. [71]. The mass fraction of platinum group metals (PGM) in the resulting material was 28.8 wt.%. The obtained material was labeled as PtPd/C.

#### 4.1.2. Synthesis of PtPd_CoNiCu/C Catalysts

A measured quantity of the PtPd/C catalyst (PGM content of 28.8 wt.%) was placed in a beaker, and a mixture of ethylene glycol (reagent grade, JSC «ECOS-1», Moscow, Russia) and bi-distilled water in a 4:1 ratio was added. A magnetic stir bar was placed into the resulting suspension, and it was stirred on a magnetic stirrer for 2–3 min. Subsequently, the mixture was dispersed ultrasonically twice for 2 min each (Ultrasonic Processor FS-1200N, Henan Chengyi Equipment Science and Technology Co., Zhengzhou, China) and returned to the magnetic stirrer. Calculated volumes of the precursor solutions—cobalt (CoSO_4_·7H_2_O), copper (CuSO_4_·7H_2_O), and nickel (NiSO_4_·7H_2_O)—as 0.05 M aqueous solutions were added using a dispenser, and stirring was continued for 1 h [26]. After this, a calculated amount of a 0.5 M NaOH solution was introduced to precipitate the hydroxides of cobalt, copper, and nickel. The mixture was again left stirring on a magnetic stirrer for 1 h. The catalyst suspension was filtered using a Büchner funnel and “blue ribbon” filter paper, followed by sequential washing with water, ethyl alcohol, and again with water, each for no less than three times. The resulting catalyst on the filter was then dried at 80 °C in a vacuum drying oven and labeled as PtPd_CoNiCu_Alk/C. After drying, the catalyst was separated from the filter paper, and the resulting powder was subjected to thermal treatment in a tube furnace at 600 °C for 1 h under a flow of inert gas containing 5% hydrogen. The final catalyst was labeled as PtPd_CoNiCu/C. The synthesis procedure is illustrated in Figure S1.

### 4.2. Catalyst Characterization Techniques

The phase composition of the synthesized materials was studied by X-ray diffraction (XRD) using an ARL X’TRA diffractometer with CuKα radiation (λ = 1.5406 Å). Scanning was performed in the 2θ range of 15–95° with a step size of 0.04° and a scanning rate of 2°/min. Elemental analysis was carried out by Total Reflection X-ray Fluorescence (TXRF) on an RFS-001 spectrometer operating in total reflection mode (Research Institute of Physics, Southern Federal University, Rostov-on-Don, Russia). Thermal analysis was performed using a combined TGA/DSC analyzer NETZSCH STA 449 C Jupiter (NETZSCH-Gerätebau GmbH, Selb, Bavaria, Germany). The experiments were carried out in an 80% N_2_/20% O_2_ atmosphere, with a temperature ramp from 40 to 800 °C at 10 °C/min, a gas flow of 20 mL/min, and using alumina crucibles. High-resolution transmission electron microscopy (HRTEM), selected area electron diffraction (SAED), high-angle annular dark field (HAADF), secondary and backscattered electron imaging (SEI/BEI) and energy dispersive X-ray spectroscopy (EDX) analyses were conducted using JEOL JEM-F200 (JEOL, Tokyo, Japan), which is operated at 200 kV accelerating voltage, equipped with a cold field-emission gun (CFEG) and high-resolution CMOS AMT camera. AMT software (AMT Capture Engine, version 7.0.1.189) was used to decipher the electron diffraction distances. TED Pella Evaporated aluminum was used as a standard to calibrate camera length for SAED. For both TEM and EDX measurements, JEOL EM-01361RSTHB double-tilt beryllium specimen holder was used. EDX was performed with Bruker Xflash 6T/60 Quantax 400-STEM system with 4000 channels, including an energy-dispersive Peltier-cooled XFlash detector, 0.45 mm detector thickness. Morphology of the catalysts was studied by transmission electron microscopy (HRTEM) and scanning transmission electron microscopy (STEM) using a Themis Z transmission electron microscope (Thermo Fisher Scientific, Breda, The Netherlands). The microscope was operated at an accelerating voltage of 200 kV that provided a resolution of 0.07 nm in the TEM mode. The microscope was also equipped with a SuperX energy-dispersive spectrometer (Thermo Fisher Scientific, The Netherlands), which was used for elemental mapping. The samples for research were fixed on aluminum grids using ultrasonic dispersion of the catalysts in ethanol for the standard experiments.

The surface chemical composition of the samples was studied by X-ray photoelectron spectroscopy (XPS) using a SPECS (Surface Nano Analysis GmbH, Berlin, Germany) photoelectron spectrometer. The spectrometer is equipped with a PHOIBOS-150-MCD-9 hemispherical analyzer and an XR-50 X-ray source with a dual Al/Mg anode. The spectra were acquired using non-monochromated Al Kα radiation (hν = 1486.6 eV). The powder catalyst samples were mounted on the sample holder using double-sided conductive adhesive tape. Data processing was performed using the CasaXPS software package, version 2.3.26.

The relative elemental concentrations were determined based on the integral intensities of the core-level spectra using the cross-sections according to Scofield. The binding energy (BE) scale was calibrated using the C 1*s* peak (BE = 284.5 eV), attributed to the graphitic (C=C) [72]. For detailed analysis, the spectra were fitted into several peaks after the background subtraction by the Shirley method. The peak shapes were approximated by a symmetric (GL) and an asymmetric Lorentzian (LF) line shapes obtained by convolution of a Lorentzian with a Gaussian.

### 4.3. Methods for Studying Catalytic Activity

The electrochemical behavior of the electrocatalysts was studied using a three-electrode cell and a rotating disk electrode (RDE) (Pine Research MSR Rotator, Durham, NC, USA) in a 0.1 M HClO_4_ electrolyte [72]. The catalyst was applied onto the surface of a glassy carbon electrode using catalytic ink. To prepare the ink, 0.004 g of the catalyst material was mixed with 50 μL of deionized H_2_O, 21 μL of a 5 wt.% Nafion^®^ solution, and 1950 μL of isopropyl alcohol. The resulting suspension was dispersed via ultrasonication for 30 min to achieve a homogeneous dispersion. An aliquot of a calculated volume was deposited onto the glassy carbon disk electrode to achieve a platinum group metal (PGM) loading of approximately 20 μg/cm^2^.

The electrode surface was standardized, and cyclic voltammograms (CVs) were recorded to determine the ECSA in accordance with the methodology described elsewhere [73,74]. The electrochemical CO oxidation (CO-stripping) was studied by purging the 0.1 M HClO_4_ deaerated solution with the CO gas for 10 min, with the working electrode held at E = 0.1 V. The cell was then purged with Ar for 30 min to remove excess CO. Next, potential was scanned in the anode direction up to 1.10 V at a sweep rate of 20 mV/s.

Catalytic performance for the oxygen reduction reaction was quantified by linear sweep voltammetry with a rotating disk electrode. The corresponding kinetic current at 0.9 V was determined using the Koutecky-Levich formalism [73,74]. All potentials are referenced to the reversible hydrogen electrode (RHE).

Accelerated stress tests were employed to assess catalyst stability, applying 10,000 square-wave potential cycles from 0.4 to 1.0 V vs. RHE (3 s per potential) in an O_2_-saturated 0.1 M HClO_4_ solution at room temperature (25 °C). Following AST, the electrode surface was conditioned by 100 cyclic voltammetry cycles (0.04–1.00 V, 200 mV/s), and the ECSA after stress-test was determined from two subsequent CV cycles.

## Figures and Tables

**Table 1:** Structural characteristics of the synthesized catalysts.

Sample	*ω* (M), %	*ω* (PMG), %	Composition by TXRF	a, Å	D_av_ Cryst., nm	D_av_ NP, nm
PtPd/C	28.8	28.8	PtPd_0.9_	3.941	1.9	-
PtPd_CoNiCu/C	36.3	19.9	Pt_16_Pd_17_Co_24_Ni_22_Cu_21_	3.752	5.5	6.3
Hispec 3000	20	20	Pt	3.923	2.5	3.0

**Table 2:** Electrochemical characteristics of the catalysts.

Sample	ECSA H_ads/des_, m^2^/g (PGM)	ECSA CO, m^2^/g (PGM)	I_mass_, A/g (PGM)	I_spec_, A/m^2^ (PGM)	E_1/2_, V
PtPd/C	83	86	146	1.8	0.89
PtPd_CoNiCu/C	63	63	269	4.3	0.90
Hispec 3000	84	-	254	3.0	0.90

## Data Availability

All data are presented in the main text and Supplementary Materials.

## Supplementary Materials

The following supporting information can be downloaded at: https://www.mdpi.com/article/10.3390/ijms262311504/s1.
