# Supplementary material for: High-Temperature Synthesis of High-Entropy Alloy PtPd_CoNiCu Nanoparticles as a Catalyst for the Oxygen Reduction Reaction
Source: Int J Mol Sci. 2025 Nov 27;26(23):11504. doi: 10.3390/ijms262311504 (PMC12692486; doi:10.3390/ijms262311504)
Supplement: Supplementary file 1 [file ijms-26-11504-s001.zip › ijms-3997860-supplementary.pdf]

Supplementary Materials

High-Temperature Synthesis of High-Entropy Alloy PtPd_CoNiCu Nanoparticles as a Catalyst for the Oxygen Reduction Reaction

AlinaNevelskaya[1,2], Anna Gavrilova[1], Nikolay Lyanguzov[3], Mikhail Tolstunov[2], Ilya Pankov[4], Anna Kremneva[5], Evgeny Gerasimov[5], Andrey Kokhanov[1], Sergey Belenov[1,*]

[1] Faculty of Chemistry, Southern Federal University, 7 Zorge St., Rostov-on-Don 344090, Russia; alya.nevelskaya@mail.ru (A.N.); agavrilo@sfedu.ru (A.G.); akokhanov@sfedu.ru (A.K.)
[2] Federal Research Center the Southern Scientific Center of the Russian Academy of Sciences (SSC RAS),
41 Chekhov Ave., Rostov-on-Don 344006, Russia; miftol@yandex.ru
[3] Department of Physics, Southern Federal University, 5 Zorge St., Rostov-on-Don 344090, Russia; nvlyanguzov@sfedu.ru
[4] Research Institute of Physical Organic Chemistry, Southern Federal University, 194/2 Stachki St.,
Rostov-on-Don 344090, Russia; ipankov@sfedu.ru
[5] Boreskov Institute of Catalysis, Ac. Lavrentieva Ave. 5, Novosibirsk 630090, Russia; kremneva@catalysis.ru (A.K.); gerasimov@catalysis.ru (E.G.)
[*] Correspondence: sbelenov@sfedu.ru; Tel.: +7-904-44-99-483

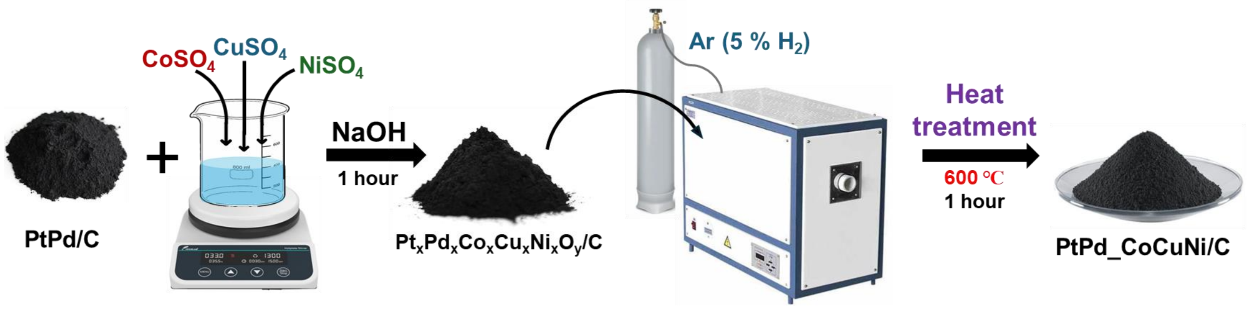

Figure S1. Scheme of high-temperature synthesis of HEA PtPd_CoNiCu/C catalyst

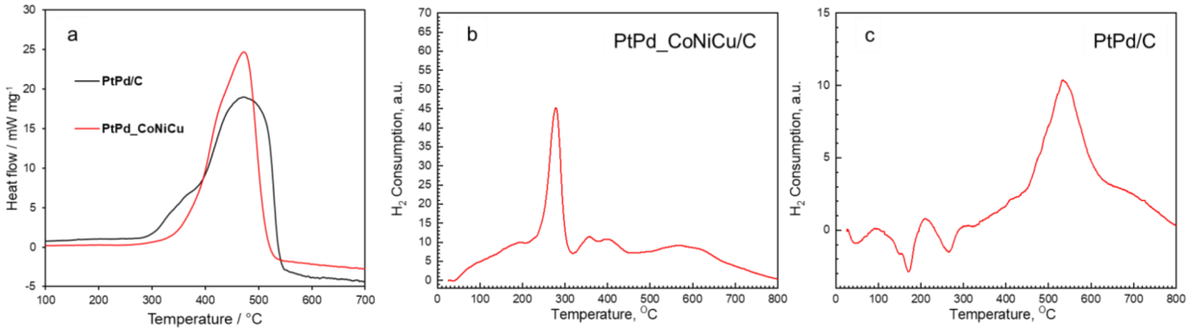

Figure S2. Results of differential scanning calorimetry (a) of PtPd/C and PtPd_CoNiCu/C catalyst and TPR of PtPd_CoNiCu/C (b) and PtPd/C (c)

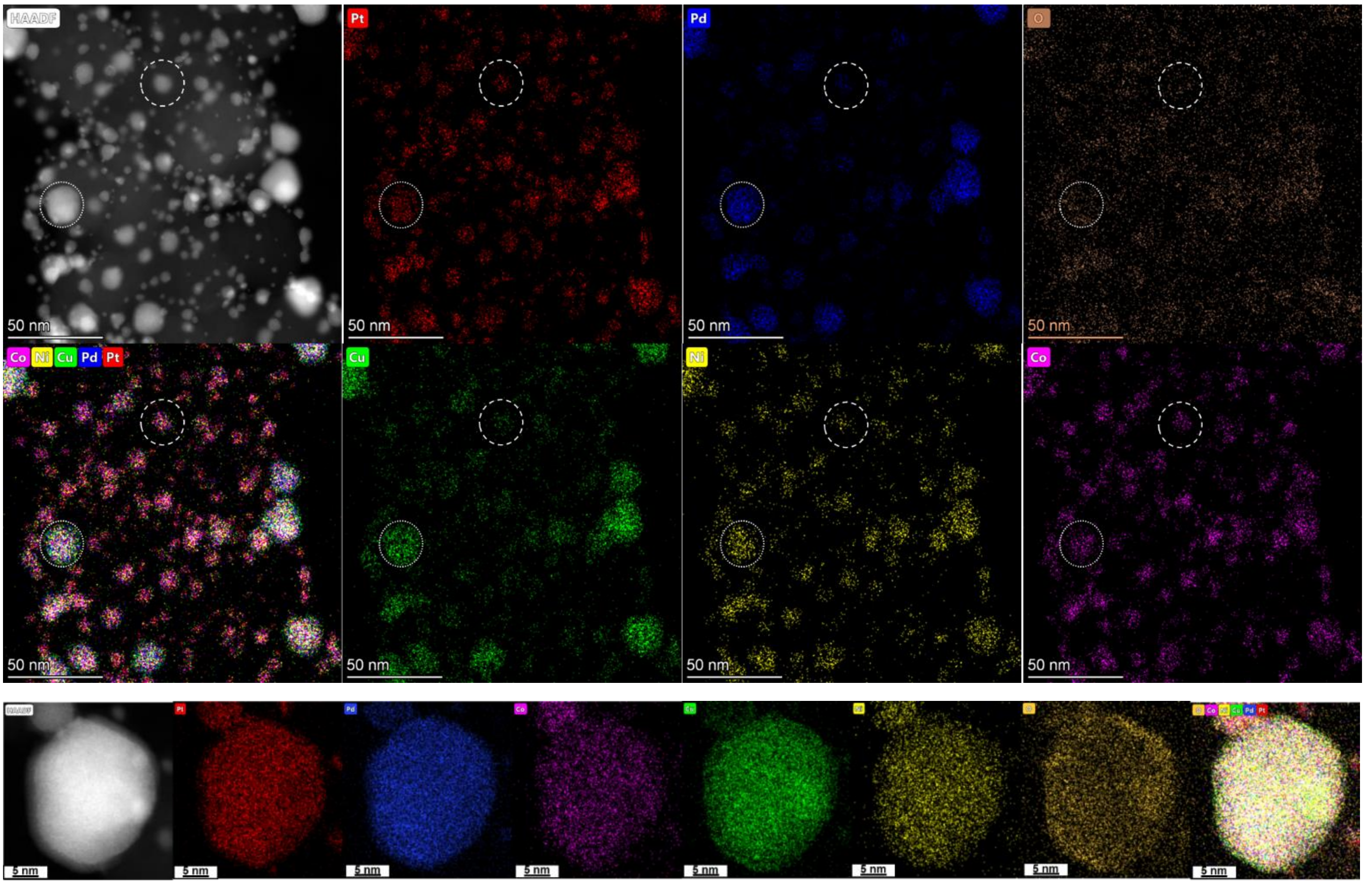

Figure S3. High-angle annular dark-field scanning transmission electron microscopy (HAADF-STEM) image and elemental mapping of this area of PtPd_CoNiCu/C catalysts. All images feature large nanoparticles outlined with a short-dashed line and small nanoparticles marked with a long-dashed line. Elemental mapping for an individual large nanoparticle approximately 15 nm in size is presented separately.

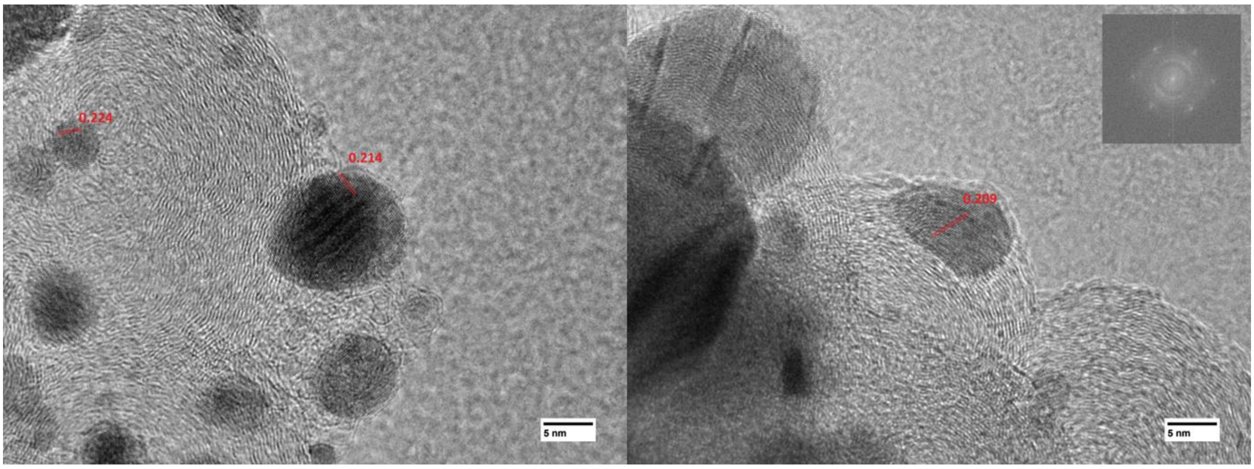

Figure S4. HRTEM images of PtPd_CoNiCu/C catalyst, insert – FFT of the selected area.

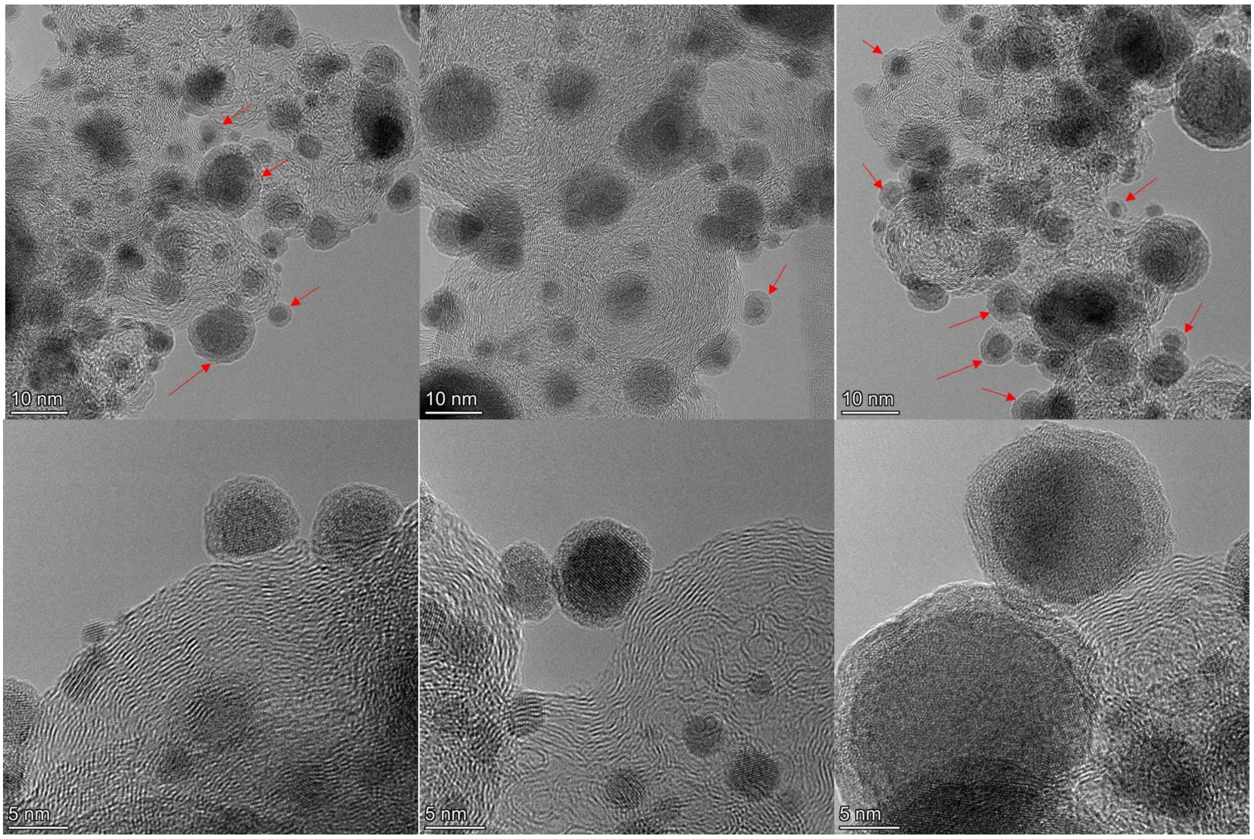

Figure S5. HRTEM images of PtPd_CoNiCu/C catalyst

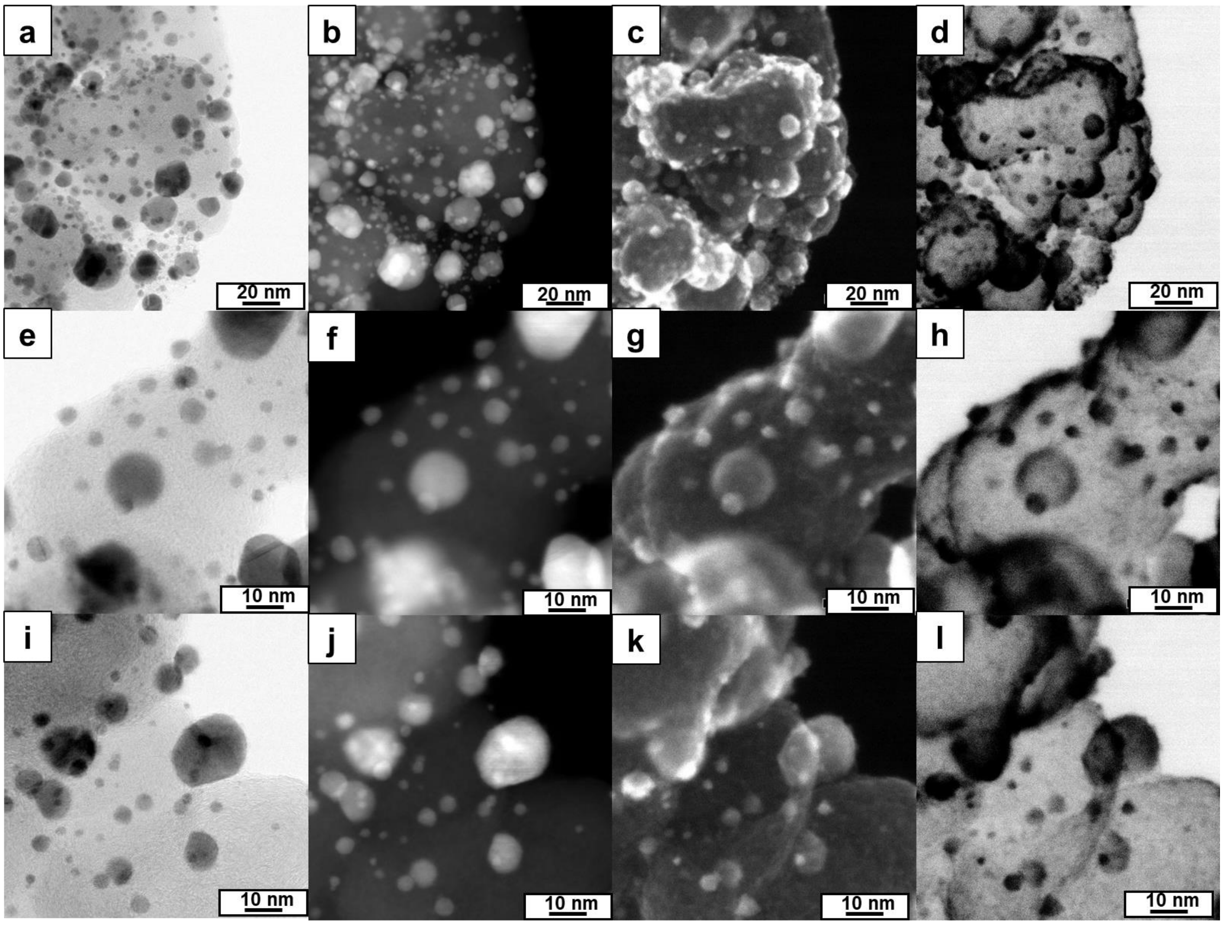

Figure S6. BF-STEM (a, e, i), HAADF (b, f, j), SEI (c, g, k), BEI (d, h, l) images of PtPd_CoNiCu/C catalyst

Table S1. Pt4$f_{7/2}$, Pd3$d_{5/2}$, Cu2$p_{3/2}$, Ni2$p_{3/2}$, Co2$p_{3/2}$, C1s and O1s. binding energies (eV) of PtPd_CoNiCu/C catalyst

C1s, eV	O1s, eV	Pt4$f_{7/2}$, eV	Pd3$d_{5/2}$, eV	Cu2$p_{3/2}$, eV (%)	Ni2$p_{3/2}$, eV (%)	Co2$p_{3/2}$, eV (%)
284.5	529.9<br>531.7<br>533.5	71.3	335.5	932.1<br>(57)<br>934.2<br>(43)	852.7<br>(27)<br>855.6<br>(73)	778.4<br>(16)<br>780.0<br>(84)
